# 2-Amino­benzothia­zolium 2,4-dicarboxy­benzoate monohydrate

**DOI:** 10.1107/S160053680901890X

**Published:** 2009-05-23

**Authors:** Na Zhang, Kai-Sheng Liu, Xiao-Jun Zhao

**Affiliations:** a College of Chemistry and Life Science, Tianjin Key Laboratory of Structure and Performance for Functional Molecule, Tianjin Normal University, Tianjin 300387, People’s Republic of China

## Abstract

Cocrystallization of 2-amino­benzothia­zole with benzene-1,2,4-tricarboxylic acid in a mixed solvent affords the title ternary cocrystal, C_7_H_7_N_2_S^+^·C_9_H_5_O_6_^−^·H_2_O, in which one of the carboxyl groups of the benzene­tricarboxylic acid is deproton­ated and the heterocyclic N atom of the 2-amino­benzothia­zole is protonated. In the crystal, inter­molecular N—H⋯O and O—H⋯O hydrogen-bonding inter­actions stabilize the packing.

## Related literature

For the properties of benzothia­zole and its derivative and their uses in crystal engineering, see: Batista *et al.* (2007 ▶); Leng *et al.* (2001 ▶); Chen *et al.* (2008 ▶); Kovalska *et al.* (2006 ▶); Marconato *et al.* (1998 ▶). For 2-amino­benzothia­zole (Abt) metal complexes, see: Batı *et al.* (2005 ▶); Sieroń & Bukowska-Strzyzewska (1999 ▶); Usman *et al.* (2003 ▶). For Abt-based cocrystals, see: Lynch *et al.* (1998 ▶, 1999 ▶).
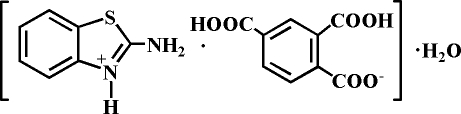


## Experimental

### 

#### Crystal data

C_7_H_7_N_2_S^+^·C_9_H_5_O_6_^−^·H_2_O
*M* *_r_* = 378.35
Orthorhombic, 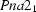

*a* = 6.8510 (4) Å
*b* = 24.3789 (15) Å
*c* = 9.7043 (6) Å
*V* = 1620.81 (17) Å^3^
*Z* = 4
Mo *K*α radiation
μ = 0.25 mm^−1^
*T* = 296 K
0.20 × 0.18 × 0.17 mm

#### Data collection

Bruker APEXII CCD area-detector diffractometer
Absorption correction: multi-scan (*SADABS*; Sheldrick, 1996 ▶) *T*_min_ = 0.953, *T*_max_ = 0.960
7728 measured reflections
2632 independent reflections
2446 reflections with *I* > 2σ(*I*)
*R*_int_ = 0.023

#### Refinement

*R*[*F* ^2^ > 2σ(*F* ^2^)] = 0.030
*wR*(*F* ^2^) = 0.070
*S* = 1.04
2632 reflections
237 parameters
1 restraint
H-atom parameters constrained
Δρ_max_ = 0.15 e Å^−3^
Δρ_min_ = −0.18 e Å^−3^
Absolute structure: Flack (1983 ▶), 1116 Friedel pairs
Flack parameter: 0.10 (8)

### 

Data collection: *APEX2* (Bruker, 2003 ▶); cell refinement: *SAINT* (Bruker, 2001 ▶); data reduction: *SAINT*; program(s) used to solve structure: *SHELXS97* (Sheldrick, 2008 ▶); program(s) used to refine structure: *SHELXL97* (Sheldrick, 2008 ▶); molecular graphics: *SHELXTL* (Sheldrick, 2008 ▶) and *DIAMOND* (Brandenburg & Berndt, 1999 ▶); software used to prepare material for publication: *SHELXL97*.

## Supplementary Material

Crystal structure: contains datablocks I, global. DOI: 10.1107/S160053680901890X/bt2963sup1.cif

Structure factors: contains datablocks I. DOI: 10.1107/S160053680901890X/bt2963Isup2.hkl

Additional supplementary materials:  crystallographic information; 3D view; checkCIF report

## Figures and Tables

**Table 1:** Hydrogen-bond geometry (Å, °)

*D*—H⋯*A*	*D*—H	H⋯*A*	*D*⋯*A*	*D*—H⋯*A*
O4—H4⋯O7^i^	0.82	1.86	2.674 (2)	171
O6—H6⋯O2^ii^	0.82	1.82	2.635 (2)	171
N1—H1⋯O1^iii^	0.86	1.85	2.698 (2)	170
N2—H2*A*⋯O3^iii^	0.86	2.03	2.838 (3)	156
N2—H2*B*⋯O5	0.86	1.95	2.776 (3)	160
O7—H7*A*⋯O1^iv^	0.85	2.00	2.851 (2)	177
O7—H7*B*⋯O2^ii^	0.85	2.05	2.891 (2)	170

Symmetry codes: (i) 
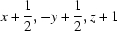
; (ii) 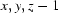; (iii) 
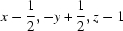
; (iv) 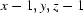.

**Fig. 1.**
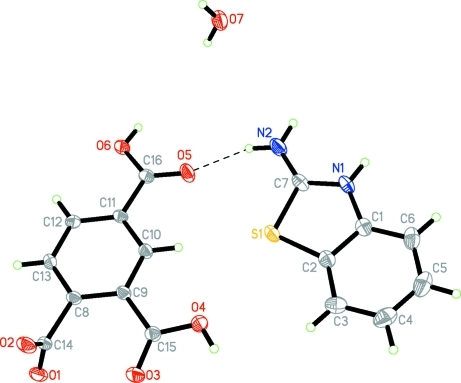
The molecular structure of (I), drawn with 30% probability displacement ellipsoids.

**Fig. 2.**
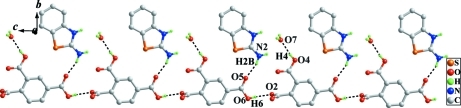
A perspective view of the one-dimensional hydrogen–bonded ribbon of (I). Hydrogen bonds are indicated by dashed lines.

**Fig. 3.**
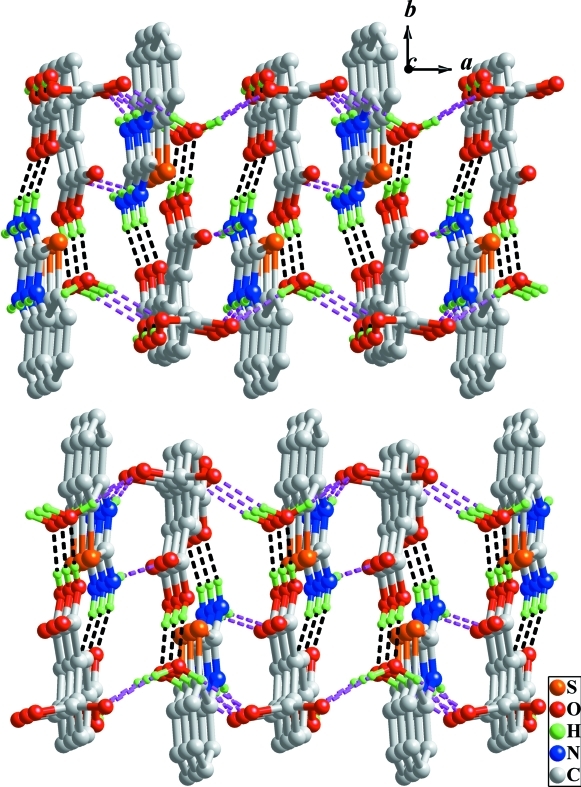
The separate two-dimensional supramolecular sheet of (I).

## supplementary crystallographic information

### Comment

Benzothiazole and its derivatives are extensively used in the field of crystal engineering owing to their beautiful structure and potential applications as electroluminescent devices (Batista *et al.*, 2007; Leng *et al.*, 2001, Chen *et al.*, 2008), fluorescent probes for DNA (Kovalska *et al.*, 2006), and corrosion inhibitors (Marconato *et al.*, 1998).

As one of the typical benzothiazole derivatives, 2-aminobenzothiazole (Abt) has been becoming a promising candidate for both the metal complexes and organic cocrystals, because they have rigid heterocyclic backbone and functional amino group. Consequently, various Abt–based metal complexes with diverse coligands have been considerably investigated (Batı *et al.*, 2005; Sieroń *et al.*, 1999; Usman *et al.*, 2003). In contrast, the Abt-based cocrystals are limited documented (Lynch *et al.*, 1998; Lynch *et al.*, 1999). Thus, as a continuation of acid–base crystalline adducts, in the present paper, we choose Abt and aromatic 1, 2, 4-benzenetricarboxylic acid (H_3_btc) as building blocks to cocrystallize. As a result, an intermolecular proton–transfer adduct, (I), was obtained, which exhibits a two–dimensional hydrogen–bonded network.

As shown in Fig. 1, the asymmetric unit of (I) comprises one HAbt cation, a monodeprontonated H_2_btc anion and one water molecule. The exocyclic amino group of HAbt is roughly coplanar with the benzothiazole ring. In contrast, the deprotonated carboxy group of H_2_btc makes dihedral angle of 86.103 (1)°, and the other carboxylic groups form dihedral angles of 8.231 (1) and 1.962 (2)° with the benzene ring of H_2_btc, respectively. The benzothiazole and the benzene rings of H_2_btc exhibits a dihedral angle of 7.083 (2)°. In the asymmetric unit, an intermolecular N2–H2B ···O5 hydrogen–bonding interaction (Table 1) was observed to stabilize the adduct.

Two H_2_btc anions from the adjacent units are held together by intermolecular O6–H6···O2 interactions (Table 1) to form an infinite one–dimensional ribbon along the crystallographic *c*–axis (Fig. 2), in which lattice water molecules was entrapped by O4–H4···O7 hydrogen–bonding interaction between the carboxylic group of H_2_btc and water molecule..

Furthermore, the neighboring 1–D ribbons are head–to–tail connected together by four fold O4–H4···O7, N1–H1···O1, N2–H2A···O3, O7–H7A···O1 and O7–H7B···O2 hydrogen-bonding to form a separate two-dimensional supramolecular sheet without any weak π···πinteractions between neighboring sheets (Fig. 3). Thus, it can be concluded that the extensive hydrogen–bonding interactions play essentially roles for the extension of (I).

### Experimental

2–Aminobenzothiazole (0.1 mmol, 15.0 mg) and 1, 2, 4–benzenetricarboxylic acid (0.1 mmol, 21.0 mg) were mixed in a CH_3_OH/H_2_O solution (v: v = 1:1, 10 ml) and stirred constantly for about 30 min. The resulting mixture was filtered. Colorless block crystals suitable for *X*–ray diffraction were collected by slow evaporation of the filtrate within one week. Yield: 56%. Anal. Calcd for C_16_H_14_N_2_O_7_S: C, 50.79; H, 3.73; N, 7.40%. Found: C, 50.66; H, 3.52; N, 7.28%.

### Refinement

H atoms were located in difference maps, but were subsequently placed in calculated positions and treated as riding, with C – H = 0.93, O – H = 0.85, and N – H = 0.86 Å and  *U*_iso_(H) = 1.2 *U*_eq_(C,N) or 1.5 *U*_eq_(O).

### Crystal data

**Table:**

C_7_H_7_N_2_S^+^·C_9_H_5_O_6_^−^·H_2_O	*D*_x_ = 1.551 Mg m^−^^3^
*M**_r_* = 378.35	Mo *K*α radiation, λ = 0.71073 Å
Orthorhombic, *P**n**a*2_1_	Cell parameters from 4384 reflections
*a* = 6.8510 (4) Å	θ = 3.1–27.9°
*b* = 24.3789 (15) Å	µ = 0.25 mm^−^^1^
*c* = 9.7043 (6) Å	*T* = 296 K
*V* = 1620.81 (17) Å^3^	Block, colourless
*Z* = 4	0.20 × 0.18 × 0.17 mm
*F*(000) = 784	

### Data collection

**Table:**

Bruker APEXII CCD area-detector diffractometer	2632 independent reflections
Radiation source: fine-focus sealed tube	2446 reflections with *I* > 2σ(*I*)
graphite	*R*_int_ = 0.023
φ and ω scans	θ_max_ = 25.0°, θ_min_ = 1.7°
Absorption correction: multi-scan (*SADABS*; Sheldrick, 1996)	*h* = −7→8
*T*_min_ = 0.953, *T*_max_ = 0.960	*k* = −29→19
7728 measured reflections	*l* = −11→8

### Refinement

**Table:**

Refinement on *F*^2^	Secondary atom site location: difference Fourier map
Least-squares matrix: full	Hydrogen site location: inferred from neighbouring sites
*R*[*F*^2^ > 2σ(*F*^2^)] = 0.030	H-atom parameters constrained
*wR*(*F*^2^) = 0.070	*w* = 1/[σ^2^(*F*_o_^2^) + (0.0386*P*)^2^ + 0.1846*P*] where *P* = (*F*_o_^2^ + 2*F*_c_^2^)/3
*S* = 1.04	(Δ/σ)_max_ = 0.001
2632 reflections	Δρ_max_ = 0.14 e Å^−^^3^
237 parameters	Δρ_min_ = −0.18 e Å^−^^3^
1 restraint	Absolute structure: Flack (1983), 1116 Friedel pairs
Primary atom site location: structure-invariant direct methods	Flack parameter: 0.10 (8)

### Special details

**Table:**

Geometry. All e.s.d.'s (except the e.s.d. in the dihedral angle between two l.s. planes) are estimated using the full covariance matrix. The cell e.s.d.'s are taken into account individually in the estimation of e.s.d.'s in distances, angles and torsion angles; correlations between e.s.d.'s in cell parameters are only used when they are defined by crystal symmetry. An approximate (isotropic) treatment of cell e.s.d.'s is used for estimating e.s.d.'s involving l.s. planes.
Refinement. Refinement of *F*^2^ against ALL reflections. The weighted *R*-factor *wR* and goodness of fit *S* are based on *F*^2^, conventional *R*-factors *R* are based on *F*, with *F* set to zero for negative *F*^2^. The threshold expression of *F*^2^ > σ(*F*^2^) is used only for calculating *R*-factors(gt) *etc*. and is not relevant to the choice of reflections for refinement. *R*-factors based on *F*^2^ are statistically about twice as large as those based on *F*, and *R*- factors based on ALL data will be even larger.

### Fractional atomic coordinates and isotropic or equivalent isotropic displacement parameters (Å^2^)

**Table:**

	*x*	*y*	*z*	*U*_iso_*/*U*_eq_	
S1	0.41587 (9)	0.29601 (2)	0.74515 (7)	0.04137 (16)	
O1	0.6936 (2)	0.09280 (7)	1.31365 (15)	0.0397 (4)	
O2	0.3712 (2)	0.10037 (7)	1.34480 (16)	0.0415 (4)	
O3	0.5680 (3)	0.20862 (7)	1.2595 (2)	0.0566 (5)	
O4	0.4844 (3)	0.25690 (7)	1.07597 (17)	0.0464 (4)	
H4	0.5055	0.2826	1.1284	0.070*	
O5	0.3884 (3)	0.17106 (7)	0.63412 (16)	0.0484 (5)	
O6	0.3848 (3)	0.08050 (6)	0.61157 (14)	0.0382 (4)	
H6	0.3681	0.0882	0.5302	0.057*	
N1	0.3226 (3)	0.35998 (8)	0.54800 (19)	0.0397 (5)	
H1	0.2880	0.3717	0.4681	0.048*	
N2	0.3039 (3)	0.26695 (9)	0.4919 (2)	0.0515 (6)	
H2A	0.2647	0.2740	0.4096	0.062*	
H2B	0.3190	0.2335	0.5180	0.062*	
C1	0.3639 (3)	0.39498 (10)	0.6569 (3)	0.0369 (5)	
C2	0.4157 (3)	0.36673 (10)	0.7762 (3)	0.0376 (6)	
C3	0.4535 (4)	0.39391 (12)	0.8975 (3)	0.0497 (7)	
H3	0.4875	0.3751	0.9773	0.060*	
C4	0.4387 (4)	0.45033 (13)	0.8957 (4)	0.0601 (8)	
H4A	0.4624	0.4699	0.9763	0.072*	
C5	0.3892 (4)	0.47875 (11)	0.7767 (4)	0.0625 (9)	
H5	0.3814	0.5168	0.7790	0.075*	
C6	0.3515 (4)	0.45135 (10)	0.6548 (3)	0.0508 (7)	
H6A	0.3191	0.4702	0.5747	0.061*	
C7	0.3406 (3)	0.30693 (10)	0.5769 (2)	0.0393 (6)	
C8	0.4935 (3)	0.10831 (9)	1.1180 (2)	0.0288 (5)	
C9	0.4830 (3)	0.16013 (9)	1.0568 (2)	0.0287 (5)	
C10	0.4490 (3)	0.16392 (9)	0.9160 (2)	0.0303 (5)	
H10	0.4394	0.1983	0.8753	0.036*	
C11	0.4293 (3)	0.11747 (10)	0.8351 (2)	0.0281 (5)	
C12	0.4424 (3)	0.06607 (10)	0.8962 (2)	0.0308 (5)	
H12	0.4298	0.0345	0.8431	0.037*	
C13	0.4741 (3)	0.06218 (9)	1.0367 (2)	0.0325 (5)	
H13	0.4826	0.0277	1.0773	0.039*	
C14	0.5236 (3)	0.10060 (9)	1.2723 (2)	0.0307 (5)	
C15	0.5155 (3)	0.21044 (9)	1.1409 (2)	0.0324 (5)	
C16	0.3977 (3)	0.12557 (10)	0.6837 (2)	0.0314 (5)	
O7	0.0300 (2)	0.15288 (7)	0.2293 (2)	0.0530 (5)	
H7A	−0.0728	0.1356	0.2522	0.080*	
H7B	0.1377	0.1409	0.2607	0.080*	

### Atomic displacement parameters (Å^2^)

**Table:**

	*U*^11^	*U*^22^	*U*^33^	*U*^12^	*U*^13^	*U*^23^
S1	0.0528 (3)	0.0398 (3)	0.0315 (3)	0.0040 (3)	−0.0078 (3)	0.0088 (3)
O1	0.0446 (9)	0.0454 (10)	0.0289 (9)	0.0028 (8)	−0.0099 (7)	−0.0020 (7)
O2	0.0454 (9)	0.0585 (11)	0.0205 (7)	−0.0054 (8)	0.0019 (7)	0.0018 (7)
O3	0.0987 (14)	0.0412 (9)	0.0299 (11)	−0.0003 (10)	−0.0185 (11)	−0.0048 (9)
O4	0.0738 (12)	0.0311 (9)	0.0343 (9)	0.0022 (9)	−0.0091 (9)	−0.0041 (8)
O5	0.0828 (14)	0.0361 (10)	0.0264 (9)	0.0095 (9)	−0.0025 (9)	0.0046 (8)
O6	0.0583 (10)	0.0387 (10)	0.0177 (8)	−0.0019 (8)	−0.0019 (7)	−0.0019 (7)
N1	0.0446 (12)	0.0445 (12)	0.0300 (11)	0.0067 (9)	−0.0005 (9)	0.0139 (9)
N2	0.0771 (16)	0.0469 (13)	0.0305 (11)	0.0143 (11)	−0.0101 (11)	0.0037 (10)
C1	0.0276 (12)	0.0414 (13)	0.0415 (14)	−0.0011 (11)	0.0045 (10)	0.0082 (11)
C2	0.0299 (11)	0.0423 (13)	0.0406 (16)	−0.0003 (10)	−0.0001 (9)	0.0062 (11)
C3	0.0397 (14)	0.0623 (18)	0.0470 (16)	−0.0023 (12)	−0.0069 (12)	−0.0050 (14)
C4	0.0449 (16)	0.0600 (19)	0.075 (2)	−0.0081 (14)	−0.0057 (14)	−0.0189 (17)
C5	0.0496 (15)	0.0393 (14)	0.099 (3)	−0.0044 (13)	0.0061 (16)	−0.0028 (17)
C6	0.0430 (15)	0.0393 (14)	0.0701 (19)	−0.0007 (12)	0.0043 (13)	0.0131 (14)
C7	0.0399 (13)	0.0484 (14)	0.0295 (13)	0.0094 (11)	0.0009 (10)	0.0085 (11)
C8	0.0320 (12)	0.0339 (12)	0.0204 (11)	−0.0010 (9)	−0.0001 (9)	−0.0003 (9)
C9	0.0322 (11)	0.0314 (12)	0.0225 (10)	0.0011 (9)	−0.0002 (9)	0.0000 (9)
C10	0.0361 (12)	0.0298 (12)	0.0249 (11)	0.0009 (9)	0.0010 (9)	0.0029 (9)
C11	0.0305 (11)	0.0348 (14)	0.0191 (10)	−0.0002 (9)	0.0009 (8)	−0.0004 (9)
C12	0.0351 (12)	0.0324 (12)	0.0248 (11)	−0.0016 (9)	−0.0019 (9)	−0.0033 (9)
C13	0.0403 (12)	0.0296 (12)	0.0278 (12)	−0.0013 (10)	−0.0011 (10)	0.0050 (9)
C14	0.0431 (13)	0.0283 (11)	0.0207 (12)	−0.0029 (9)	−0.0024 (10)	−0.0006 (9)
C15	0.0383 (13)	0.0329 (12)	0.0261 (12)	0.0022 (9)	0.0007 (10)	−0.0019 (9)
C16	0.0319 (13)	0.0364 (13)	0.0259 (12)	0.0012 (10)	0.0006 (9)	−0.0007 (10)
O7	0.0487 (10)	0.0494 (10)	0.0609 (13)	0.0003 (8)	0.0007 (10)	0.0200 (10)

### Geometric parameters (Å, °)

**Table:**

S1—C7	1.733 (2)	C3—H3	0.9300
S1—C2	1.750 (2)	C4—C5	1.389 (5)
O1—C14	1.246 (3)	C4—H4A	0.9300
O2—C14	1.259 (3)	C5—C6	1.382 (4)
O3—C15	1.207 (3)	C5—H5	0.9300
O4—C15	1.313 (3)	C6—H6A	0.9300
O4—H4	0.8200	C8—C13	1.380 (3)
O5—C16	1.211 (3)	C8—C9	1.398 (3)
O6—C16	1.306 (3)	C8—C14	1.523 (3)
O6—H6	0.8200	C9—C10	1.389 (3)
N1—C7	1.329 (3)	C9—C15	1.490 (3)
N1—C1	1.388 (3)	C10—C11	1.385 (3)
N1—H1	0.8600	C10—H10	0.9300
N2—C7	1.301 (3)	C11—C12	1.389 (3)
N2—H2A	0.8600	C11—C16	1.498 (3)
N2—H2B	0.8600	C12—C13	1.384 (3)
C1—C6	1.377 (3)	C12—H12	0.9300
C1—C2	1.393 (3)	C13—H13	0.9300
C2—C3	1.376 (4)	O7—H7A	0.8502
C3—C4	1.379 (4)	O7—H7B	0.8498
			
C7—S1—C2	90.61 (12)	N1—C7—S1	112.06 (18)
C15—O4—H4	109.5	C13—C8—C9	119.23 (19)
C16—O6—H6	109.5	C13—C8—C14	118.3 (2)
C7—N1—C1	114.8 (2)	C9—C8—C14	122.44 (19)
C7—N1—H1	122.6	C10—C9—C8	119.1 (2)
C1—N1—H1	122.6	C10—C9—C15	120.6 (2)
C7—N2—H2A	120.0	C8—C9—C15	120.23 (19)
C7—N2—H2B	120.0	C11—C10—C9	121.3 (2)
H2A—N2—H2B	120.0	C11—C10—H10	119.3
C6—C1—N1	126.2 (2)	C9—C10—H10	119.3
C6—C1—C2	121.4 (2)	C10—C11—C12	119.3 (2)
N1—C1—C2	112.4 (2)	C10—C11—C16	117.6 (2)
C3—C2—C1	121.4 (2)	C12—C11—C16	123.2 (2)
C3—C2—S1	128.4 (2)	C13—C12—C11	119.5 (2)
C1—C2—S1	110.14 (19)	C13—C12—H12	120.2
C2—C3—C4	117.1 (3)	C11—C12—H12	120.2
C2—C3—H3	121.5	C8—C13—C12	121.5 (2)
C4—C3—H3	121.5	C8—C13—H13	119.3
C3—C4—C5	121.7 (3)	C12—C13—H13	119.3
C3—C4—H4A	119.1	O1—C14—O2	126.5 (2)
C5—C4—H4A	119.1	O1—C14—C8	117.49 (18)
C6—C5—C4	121.1 (3)	O2—C14—C8	115.9 (2)
C6—C5—H5	119.5	O3—C15—O4	122.5 (2)
C4—C5—H5	119.5	O3—C15—C9	122.5 (2)
C1—C6—C5	117.3 (3)	O4—C15—C9	115.03 (19)
C1—C6—H6A	121.4	O5—C16—O6	123.6 (2)
C5—C6—H6A	121.4	O5—C16—C11	121.2 (2)
N2—C7—N1	125.3 (2)	O6—C16—C11	115.1 (2)
N2—C7—S1	122.65 (19)	H7A—O7—H7B	117.1
			
C7—N1—C1—C6	−178.2 (2)	C14—C8—C9—C15	4.4 (3)
C7—N1—C1—C2	−0.3 (3)	C8—C9—C10—C11	−1.3 (3)
C6—C1—C2—C3	1.1 (4)	C15—C9—C10—C11	176.37 (19)
N1—C1—C2—C3	−177.0 (2)	C9—C10—C11—C12	0.5 (3)
C6—C1—C2—S1	179.4 (2)	C9—C10—C11—C16	−178.6 (2)
N1—C1—C2—S1	1.4 (2)	C10—C11—C12—C13	0.2 (3)
C7—S1—C2—C3	176.6 (2)	C16—C11—C12—C13	179.2 (2)
C7—S1—C2—C1	−1.59 (18)	C9—C8—C13—C12	−0.7 (3)
C1—C2—C3—C4	−0.3 (4)	C14—C8—C13—C12	178.6 (2)
S1—C2—C3—C4	−178.29 (19)	C11—C12—C13—C8	−0.1 (3)
C2—C3—C4—C5	−0.4 (4)	C13—C8—C14—O1	84.9 (3)
C3—C4—C5—C6	0.4 (4)	C9—C8—C14—O1	−95.7 (3)
N1—C1—C6—C5	176.7 (2)	C13—C8—C14—O2	−92.1 (2)
C2—C1—C6—C5	−1.1 (4)	C9—C8—C14—O2	87.3 (3)
C4—C5—C6—C1	0.4 (4)	C10—C9—C15—O3	−171.0 (2)
C1—N1—C7—N2	178.0 (2)	C8—C9—C15—O3	6.6 (3)
C1—N1—C7—S1	−1.0 (3)	C10—C9—C15—O4	8.4 (3)
C2—S1—C7—N2	−177.5 (2)	C8—C9—C15—O4	−173.95 (19)
C2—S1—C7—N1	1.47 (18)	C10—C11—C16—O5	−0.5 (3)
C13—C8—C9—C10	1.4 (3)	C12—C11—C16—O5	−179.5 (2)
C14—C8—C9—C10	−178.0 (2)	C10—C11—C16—O6	178.40 (19)
C13—C8—C9—C15	−176.3 (2)	C12—C11—C16—O6	−0.6 (3)

### Hydrogen-bond geometry (Å, °)

**Table:**

*D*—H···*A*	*D*—H	H···*A*	*D*···*A*	*D*—H···*A*
O4—H4···O7^i^	0.82	1.86	2.674 (2)	171
O6—H6···O2^ii^	0.82	1.82	2.635 (2)	171
N1—H1···O1^iii^	0.86	1.85	2.698 (2)	170
N2—H2A···O3^iii^	0.86	2.03	2.838 (3)	156
N2—H2B···O5	0.86	1.95	2.776 (3)	160
O7—H7A···O1^iv^	0.85	2.00	2.851 (2)	177
O7—H7B···O2^ii^	0.85	2.05	2.891 (2)	170

Symmetry codes: (i) *x*+1/2, −*y*+1/2, *z*+1; (ii) *x*, *y*, *z*−1; (iii) *x*−1/2, −*y*+1/2, *z*−1; (iv) *x*−1, *y*, *z*−1.
